# Laterally spreading iron, humic-like dissolved organic matter and nutrients in cold, dense subsurface water of the Arctic Ocean

**DOI:** 10.1038/srep06775

**Published:** 2014-10-27

**Authors:** Nanako Hioki, Kenshi Kuma, Yuichirou Morita, Ryouhei Sasayama, Atsushi Ooki, Yoshiko Kondo, Hajime Obata, Jun Nishioka, Youhei Yamashita, Shigeto Nishino, Takashi Kikuchi, Michio Aoyama

**Affiliations:** 1 Faculty of Fisheries Sciences, Hokkaido University, Hakodate 041-8611, Japan; 2 National Institute of Polar Research, Tachikawa 190-8518, Japan; 3 Atmosphere and Ocean Research Institute, The University of Tokyo, Kashiwa 277-8564, Japan; 4 Institute of Low Temperature Science, Hokkaido University, Sapporo 060-0189, Japan; 5 Faculty of Environmental Earth Science, Hokkaido University, Sapporo 060-0810, Japan; 6 Research Institute for Global Change, Japan Agency for Marine-Earth Science and Technology, Yokosuka 237-0061, Japan; 7 Institute of Environmental Radioactivity, Fukushima University, Fukushima 960-1296, Japan

## Abstract

The location and magnitude of oceanic iron sources remain uncertain owing to a scarcity of data, particularly in the Arctic Ocean. The formation of cold, dense water in the subsurface layer of the western Arctic Ocean is a key process in the lateral transport of iron, macronutrients, and other chemical constituents. Here, we present iron, humic-like fluorescent dissolved organic matter, and nutrient concentration data in waters above the continental slope and shelf and along two transects across the shelf–basin interface in the western Arctic Ocean. We detected high concentrations in shelf bottom waters and in a plume that extended in the subsurface cold dense water of the halocline layer in slope and basin regions. At *σ*_θ_ = 26.5, dissolved Fe, humic-like fluorescence intensity, and nutrient maxima coincided with N* minima (large negative values of N* indicate significant denitrification within shelf sediments). These results suggest that these constituents are supplied from the shelf sediments and then transported laterally to basin regions. Humic dissolved organic matter probably plays the most important role in the subsurface maxima and lateral transport of dissolved Fe in the halocline layer as natural Fe-binding organic ligand.

The surface mixed layer in the Arctic Ocean has a seasonally low salinity signature in the summer as a result of sea ice melting and river runoff. Below the surface layer, the subsurface water is dominated by a strong cold halocline. In the western Arctic Ocean, the halocline has historically been divided into an upper and a lower halocline^1^, which have a Pacific origin and an Atlantic origin, respectively. In the western Arctic Ocean, the upper halocline layer (HL) is mainly confined to the Canada Basin, and it is associated with prominent nutrient and dissolved organic matter (DOM) maxima^1^,^2^,^3^,^4^,^5^,^6^, which result from the mineralization of organic matter by interactions with the bottom sediments on the shelves after sea ice formation and brine production in the fall and winter^7^,^8^,^9^,^10^. In 1981 the upper HL was observed to extend horizontally as far as the North Pole, although its horizontal extent varies among years^7^,^10^.

The Arctic Ocean is being physically and biochemically affected by recent marine environmental changes, with the rapid decrease in summer sea ice coverage being the most pronounced^11^,^12^. The loss of seasonal and permanent sea ice cover can alter the depth of vertical mixing, the degree of stratification, light penetration, and the nutrient supply, all of which greatly influence phytoplankton bloom patterns and productivity^13^,^14^,^15^. Ecosystems in marginal seas are close to terrestrial and continental shelf sources of iron, and the supply of iron from shelf sediments to surface waters promotes high productivity in surface ecosystems^16^,^17^,^18^. In this study, we measured the distributions of iron, humic-like fluorescent DOM [as humic-like fluorescence intensity (humic F-intensity)], and nutrients in waters above the continental slope, along two transects across the shelf–basin interface, and above the continental shelf of the western Arctic Ocean (Fig. 1) and showed that these constituents are supplied from shelf sediments and then spread laterally to basin regions.

## Results and Discussion

**Figure 1:**
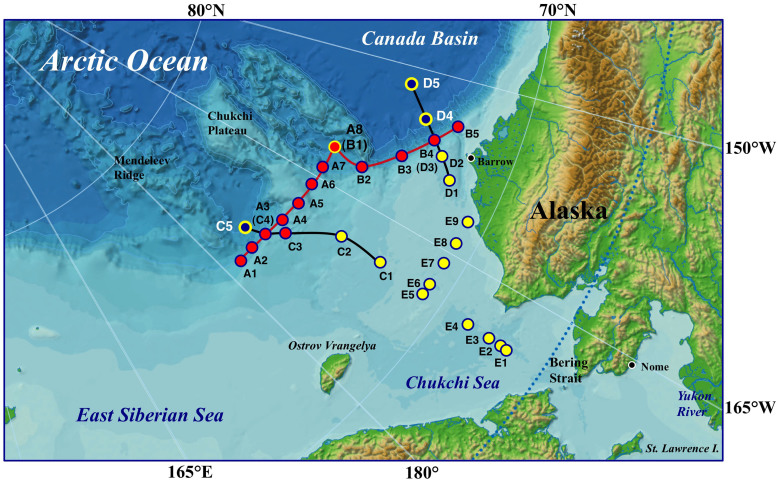
Sampling locations in the western Arctic Ocean (Chukchi Sea and Canada Basin) during 15 September to 4 October 2012. Lines A and B extend along the continental slope, and lines C and D are transects across the shelf–basin interface. Station locations: basin region, C5, D4, and D5; slope region, A1–A8(B1) (A line), A8(B1)–B5 (B line), C3; and shelf region, C1, C2, D1, D2, E1–E9. Map in this figure was created using the map “Arctic Region”, which the copyright belongs to the Japan Consortium for Arctic Environmental Research (JCAR) and the National Institute of Polar Research (NIPR). This map is open on the public via the JCAR and NIPR websites (http://www.jcar.org/menu05/01.html; http://www.nipr.ac.jp/aerc/).

**Figure 2:**
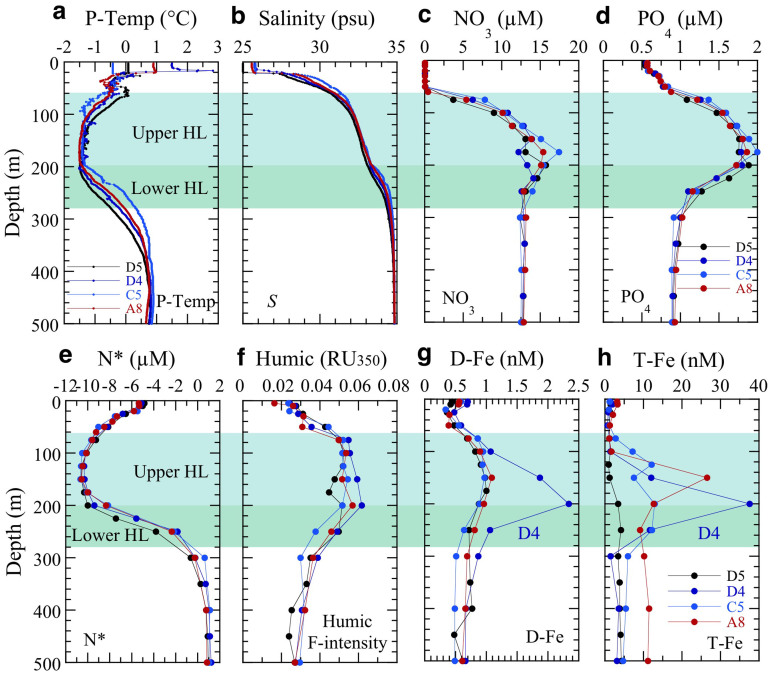
Vertical profiles of water properties and chemical constituents at typical offshore stations (A8, C5, D4, and D5). (a), (b), Potential temperature (P-Temp) (a) and salinity (b) reveal a cold halocline layer (HL); the upper HL is from ~60 to 200 m depth, and the lower HL is from ~200 to 280 m depth. (c), NO_3_. (d), PO_4_. (e), N* (an index of denitrification/anammox defined as ([NO_3_^−^] + [NO_2_^−^] + [NH_4_^+^] – 16[PO_4_^3−^] + 2.9) × 0.87 in this study to detect deviations in the [NO_3_ + NO_2_ + NH_4_]:[PO_4_] ratio from the ratio expected from the internal N cycle, given Redfield stoichiometry^19^,^20^). (f), humic F-intensity [converted to the unified scale of fluorescence Raman units at an excitation wavelength of 350 nm (RU_350_)^51^,^52^]. (g), D-Fe. (h), T-Fe.

In the surface layer at offshore stations A8, C5, D4, and D5 (Figs 1 and 2), the nitrate concentrations were extremely low regardless of the phosphate level, while the concentrations of dissolved Fe (D-Fe) and total Fe (T-Fe) were relatively high. In the surface layer, the low nitrate concentrations are probably attributable to the inflow of Bering shelf water with relatively low N* values and the biological utilization during the inflow of the Pacific water into the Arctic Ocean through the Bering Strait. Here, N* is the deviation of the nitrogen concentration from the stoichiometric relationship between nitrogenous nutrients and phosphate, and large negative values indicate the occurrence of significant denitrification within the shelf sediments^19^,^20^. In contrast, the high Fe is probably attributable to high Fe inputs from rivers^21^ and melting ice into nutrient-depleted surface waters. Denitrification, in which nitrate instead of oxygen is consumed during the bacterial decomposition of organic matter in low-oxygen waters in sediment pores, causes N* to decrease independently of the phosphate level. Generally, negative N* in the ocean interior indicates a net loss of nitrate, and the most likely cause is denitrification. Moreover, it is well known that the denitrification occurs in the bottom sediments of the broad shelves of the Okhotsk, Bering, and/or Chukchi seas^20^,^22^,^23^,^24^. Recently, it has been reported that anammox (anaerobic oxidation of ammonium using nitrite to nitrogen gas) is also an important sedimentary process decreasing N* in shallow waters of the Bering shelf and Arctic Ocean^25^,^26^.

**Figure 3:**
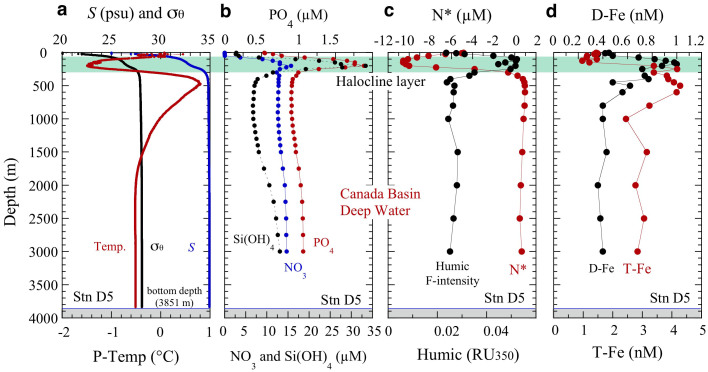
Vertical profiles of water properties and chemical constituents at a typical basin station (D5). (a), Potential temperature (P-Temp), salinity (*S*) and potential density (*σ*_θ_) reveal a cold halocline layer (HL) from ~100 to 300 m depth. (b), nutrients (NO_3_, PO_4_, and Si(OH)_4_. (c), N* and humic F-intensity. (d), D-Fe and T-Fe.

Within a narrow depth range (75–260 m) in the subsurface water at the offshore stations (Figs 2 and 3), nutrient, humic F-intensity and Fe were remarkably high, while N* values were remarkably low. The subsurface maxima and N* minima were detected in the upper HL (Figs 2c–h and 3b–d). The subsurface maxima of humic F-intensity and D-Fe (humic F-intensity, 0.05–0.06 RU_350_; D-Fe, 1–2.3 nM; T-Fe, 4–38 nM) were associated with prominent nutrient maxima (NO_3_, 13–18 µM; PO_4_, 1.7–2.0 µM; Si(OH)_4_, 15–33 µM) and N* minima (–10 to –11 µM); similar values have been reported in this depth range by recent studies^2^,^3^,^7^,^27^. Total Fe concentrations in the HL (Fig. 2h) differed markedly among stations. In contrast, vertical profiles of nutrient and D-Fe concentrations except D-Fe at station D4, N*, and humic F-intensity were generally similar among stations (Fig. 2c–g). At basin station D5 (the farthest offshore station, Fig. 1), T-Fe maxima (3.8–4.3 nM: the lowest T-Fe maxima among the slope and basin stations, Figs 2h and 3d) were found at 350–600 m below the HL even though the subsurface D-Fe maxima were in the upper HL. Therefore, the mid-depth T-Fe maxima at D5 can be attributed mainly to the downward removal of particulate Fe ([P-Fe] = [T-Fe] – [D-Fe]) from water by aggregation and particle scavenging during lateral Fe transport from shelf to basin. In deep water below about 800 m, known as the Canada Basin Deep Water (CBDW), at basin station D5, nutrient concentrations slightly increased with depth but were lower than the subsurface maximum values in the upper HL, whereas humic F-intensity, N*, and Fe concentrations were remarkably uniform. The humic F-intensity and Fe values were lower and the N* values were higher throughout the deep-water column, compared with their values in the upper part of the water column (Fig. 3). The D-Fe concentrations in CBDW were relatively constant (~0.4–0.5 nM) and similar to those in CBDW that we reported previously^2^ and to those in the deep waters of the Amundsen and Nansen basins in the central Arctic Ocean^28^.

**Figure 4:**
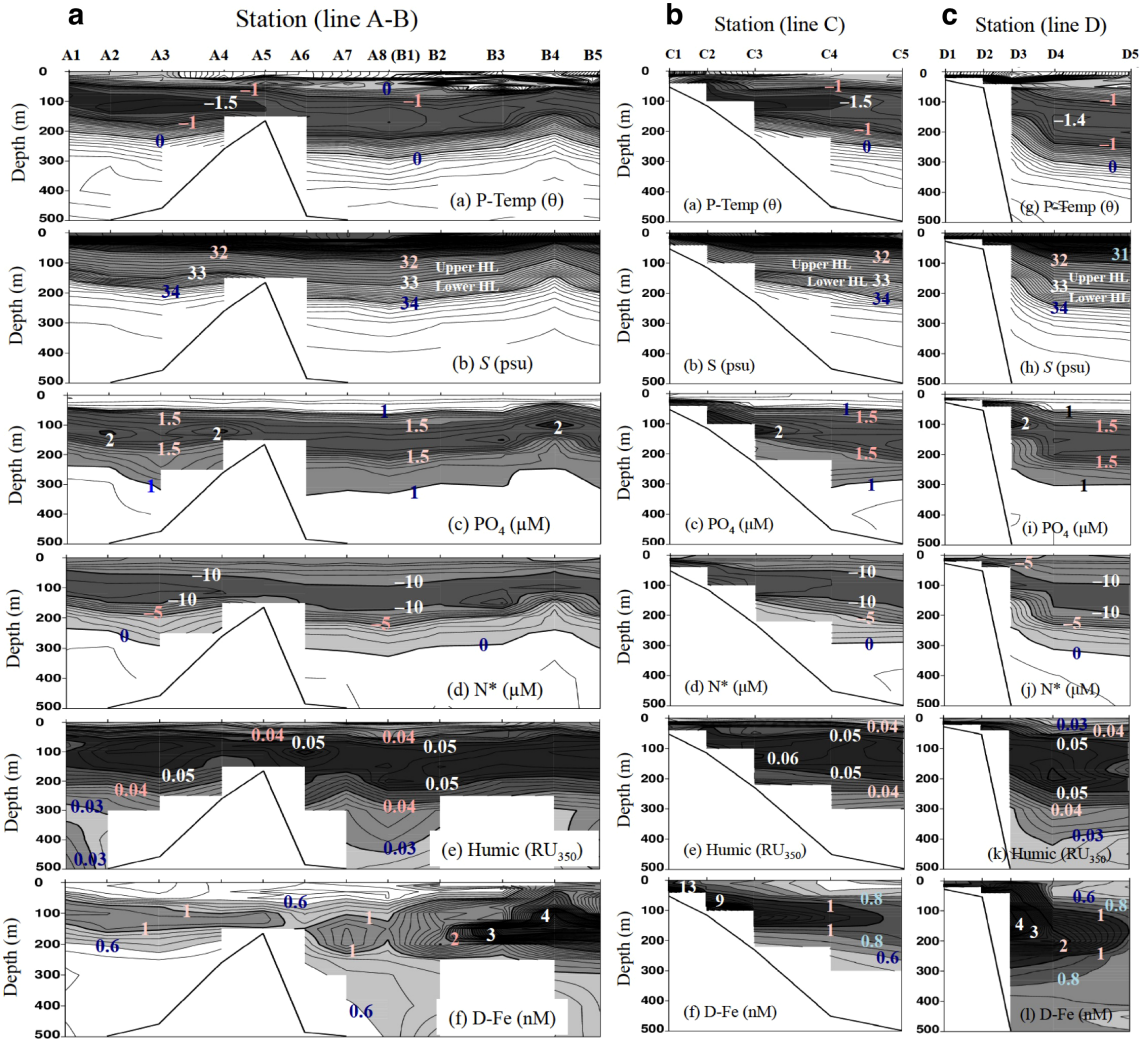
Zonal sections of water properties and chemical constituents along the slope and across the shelf–basin interface in the western Arctic Ocean. (a,b,c) Water properties (potential temperature and salinity) and chemical constituents (PO_4_, N*, humic F-intensity, and D-Fe) (a), along the slope (lines A and B, Fig. 1) and across the shelf–basin interface ((b), line C; (c), line D; Fig. 1).

In vertical section, chemical constituent distributions along the shelf slope [lines A and B: A1–A8(B1)–B5, Fig. 1] show that subsurface maxima of nutrient concentrations, humic F-intensity (0.05–0.06 RU_350_), and D-Fe (1–5 nM) concentrations and subsurface N* minima were widespread in the upper HL (Fig. 4a). Moreover, the concentration plumes along the transects across the shelf–basin interface (lines C and D: C1–C5 and D1–D5, Fig. 1) appeared to extend in the upper HL toward the basin (Fig. 4b,c). Nutrient and humic F-intensity maxima and N* minima values were nearly uniform in the upper HL (Fig. 4). However, maximum D-Fe concentrations in the upper HL, which varied from 1 to 5 nM along the slope, were remarkably high from B3 to B5 (Fig. 4a), and they decreased rapidly from inshore to offshore (Fig. 4b,c). The differing T-Fe values among stations (Fig. 2h), the mid-depth T-Fe maxima below the HL at D5 (Fig. 3d), and the rapid decrease in D-Fe values from inshore to offshore in the upper HL (Fig. 4b,c) suggest that scavenging processes limit the amount of the D-Fe transport from shelf to basin.

**Figure 5:**
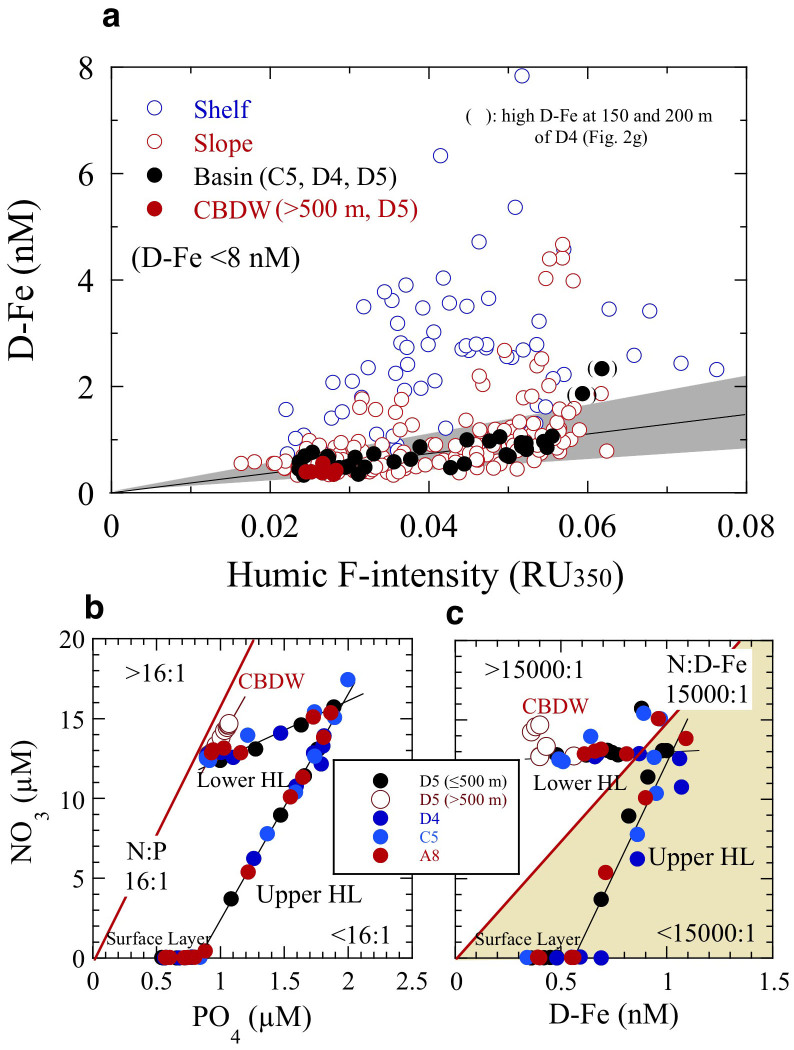
Relationships among D-Fe, humic F-intensity, and nutrients in the western Arctic Ocean. (a), D-Fe (<8 nM) versus humic F-intensity in the shelf, slope, and basin regions. The solid line indicates the Fe(III) hydroxide solubility as estimated by fitting a linear equation to the relationship between Fe(III) hydroxide solubility and humic F-intensity in the central North Pacific Ocean [Fe(III) hydroxide solubility (nM) = 18.87 ± 8.91 × humic F-intensity (RU_350_) – 0.045; *R* = 0.78, *n* = 14; ref. 30]. The grey shading area indicates within the confidence limits for a slope of the estimated Fe(III) hydroxide solubility – humic F-intensity relationship line. (b), NO_3_ versus PO_4_ at typical offshore stations (A8, C5, D4, and D5; Fig. 1). The N:P ratio is markedly low (<16:1) in the surface and HL. N:P is close to 16:1 (red line) in deep waters below the HL. (c), NO_3_ versus D-Fe at typical offshore stations. Sufficient D-Fe is present in the surface and upper HL to allow full utilization of NO_3_ by phytoplankton (N:D-Fe < 15,000:1) (ref. 38). CBDW, Canada Basin Deep Water.

Subsurface D-Fe maxima in the upper HL of the slope and basin regions, especially far from the shelf, may be maintained primarily by complexation of D-Fe with organic Fe-binding ligands such as humic DOM, which controls the Fe(III) hydroxide solubility in seawater^2^,^29^,^30^,^31^,^32^ (Fig. 5a). However, the D-Fe concentrations in the shelf region are remarkably high with respect to the Fe(III) solubility, because they plot above the estimated Fe(III) hydroxide solubility (<0.025 µm pore size) – humic F-intensity relationship line (Fig. 5a). The excess D-Fe concentrations in the shelf region are probably due to the presence of colloidal Fe (both colloidal Fe(III) hydroxide and colloidal organic matter) in the D-Fe fraction (<0.22 µm pore size), supplied from the shelf sediment. At the basin stations C5, D4, and D5 (Fig. 2g), the D-Fe concentrations in the water column plot nearly on the Fe(III) hydroxide solubility – humic F-intensity line (Fig. 5a, except for two high values at D4), indicating that humic-like fluorescent DOM controls the Fe(III) solubility and D-Fe concentrations in the basin region. Thus, the decrease in D-Fe concentrations from the shelf region to the slope and basin regions (Fig. 4b,c) can be explained by the removal of colloidal Fe from the water by aggregation and particle scavenging^2^,^29^,^30^,^33^. The D-Fe concentrations above the Fe(III) hydroxide solubility in the HL at D4, which are higher than those at A8 (B1), C5, and D5 (Figs 2g and 5a), are inferred to reflect a balance in the interplay between input and removal processes within this water mass^3^,^30^. Several types of Fe organic complexes in seawater ranged from high affinity siderophores present at low concentrations to weaker but more abundant less well-defined organic compounds such as humic substances (HS) and exopolysaccharides (EPS) with complex molecules^2^,^30^,^34^,^35^,^36^. The HS fraction is quite refractory and persists into the deep ocean, while EPS is likely to be produced in surface waters as it is associated with phytoplankton productivity. However, it is still poorly characterized for the association between Fe, HS and EPS, and the contribution of HS and EPS to the ambient ligand pool in the Arctic Ocean.

In the present study, we attributed the shoaling of the HL accompanied by high [D-Fe] and nutrients, observed at slope station B4 in the vicinity of Barrow Canyon (Figs 1 and 4a), to upwelling caused by a eddy, meander, winds, or vertical mixing^18^,^27^,^37^. This upwelling resulted in higher surface salinity (28.5–28.7), D-Fe (~1.5 nM), Si(OH)_4_ (~7 µM), and humic F-intensity (~0.035 RU_350_), despite the complete biological consumption of surface N at B4 than at other slope and basin stations (Figs 2 and 4a). In addition, the upward transport of subsurface waters enriched with iron and nutrients to the surface at B4 contributed to the surface chlorophyll *a* concentrations (0.6–0.8 µg L^−1^) being two to ten times those at other slope and basin stations. Because Fe is preferentially scavenged from the water column during the mineralization cycle, upwelled water is generally deficient in Fe compared with nitrogen species. Therefore, an additional input of Fe to surface waters is needed to reestablish the biologically required N:D-Fe balance. The maximum stoichiometric N:D-Fe mole ratio that allows complete consumption of N is 15,000:1 (ref. 38); this value was calculated by assuming a limiting C:Fe ratio in phytoplankton of 100,000:1 (ref. 39) and a C:N ratio of 6.7:1. Although we observed remarkably low N:P ratios (≪16:1) in the surface layer and HL at offshore stations (Fig. 5b), we found an N:D-Fe ratio of ~15,000 in the subsurface maximum zone of NO_3_ and D-Fe in the upper HL and of ~17,500–25,000 below the lower HL (≥300 m) at offshore stations (except for the two higher D-Fe concentrations at D4, Figs 2g and 5c). These ratios imply that the D-Fe concentration is generally sufficient to allow full utilization of NO_3_ in the upper HL.

**Figure 6:**
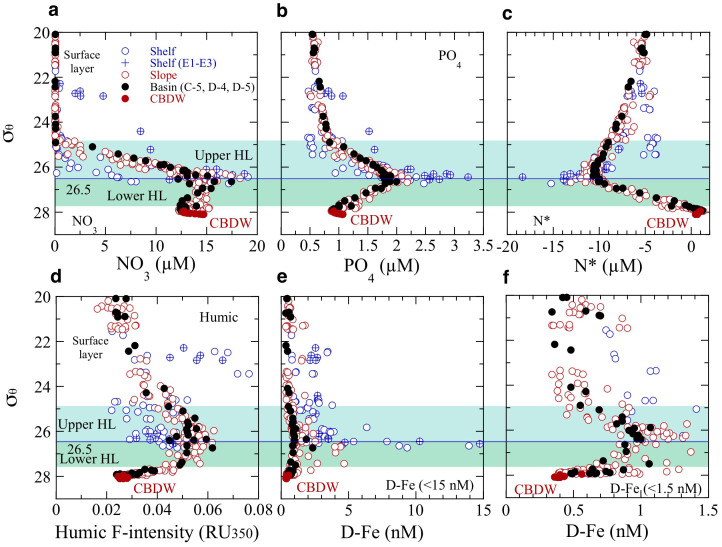
Lateral transport of chemical constituents in the halocline layer. (a,b,c,d,e,f), Potential density (*σ*_θ_) versus concentrations of chemical constituents: NO_3_ (a); PO_4_ (b); N* (c); humic F-intensity (d); D-Fe < 15 nM (e); and D-Fe < 1.5 nM (f).

Profiles of nutrients, N*, humic F-intensity, and D-Fe against potential density (*σ*_θ_) showed a well-developed halocline in the 25–27.5 *σ*_θ_ range (Fig. 6). In the shelf region, the maximum density (*σ*_θ_ = ~26.5) was observed in the bottom waters (Figs 4b,c, and 6). The fact that the maximum concentrations of nutrients and D-Fe occurred at *σ*_θ_ = 26.5 in the shelf, slope, and basin regions (Fig. 6) strongly suggests the lateral transport of these chemical constituents from the cold dense bottom waters of the shelf to the halocline in the slope and basin regions. High nutrient and D-Fe concentrations and low N* and humic-F intensity were observed in the bottom water (*T* ≤ 1°C, *S* = 33.0–33.1, *σ*_θ_ = ~26.5, Fig. 6) at the shelf stations (E1–E3) close to the Bering Strait (Fig. 1). The Bering Strait is characterized by a strongly advective physical regime that consists of three water masses (Anadyr Water, Bering Shelf Water, and Alaskan Coastal Water) flowing northward from the Bering Sea to the Chukchi Sea. The bottom water at E1–E3 is probably dominated by the nutrient-enriched Anadyr Water, which is generally saltier and colder than the Bering Shelf Water or the warm nutrient-depleted Alaskan Coastal Water.

**Figure 7:**
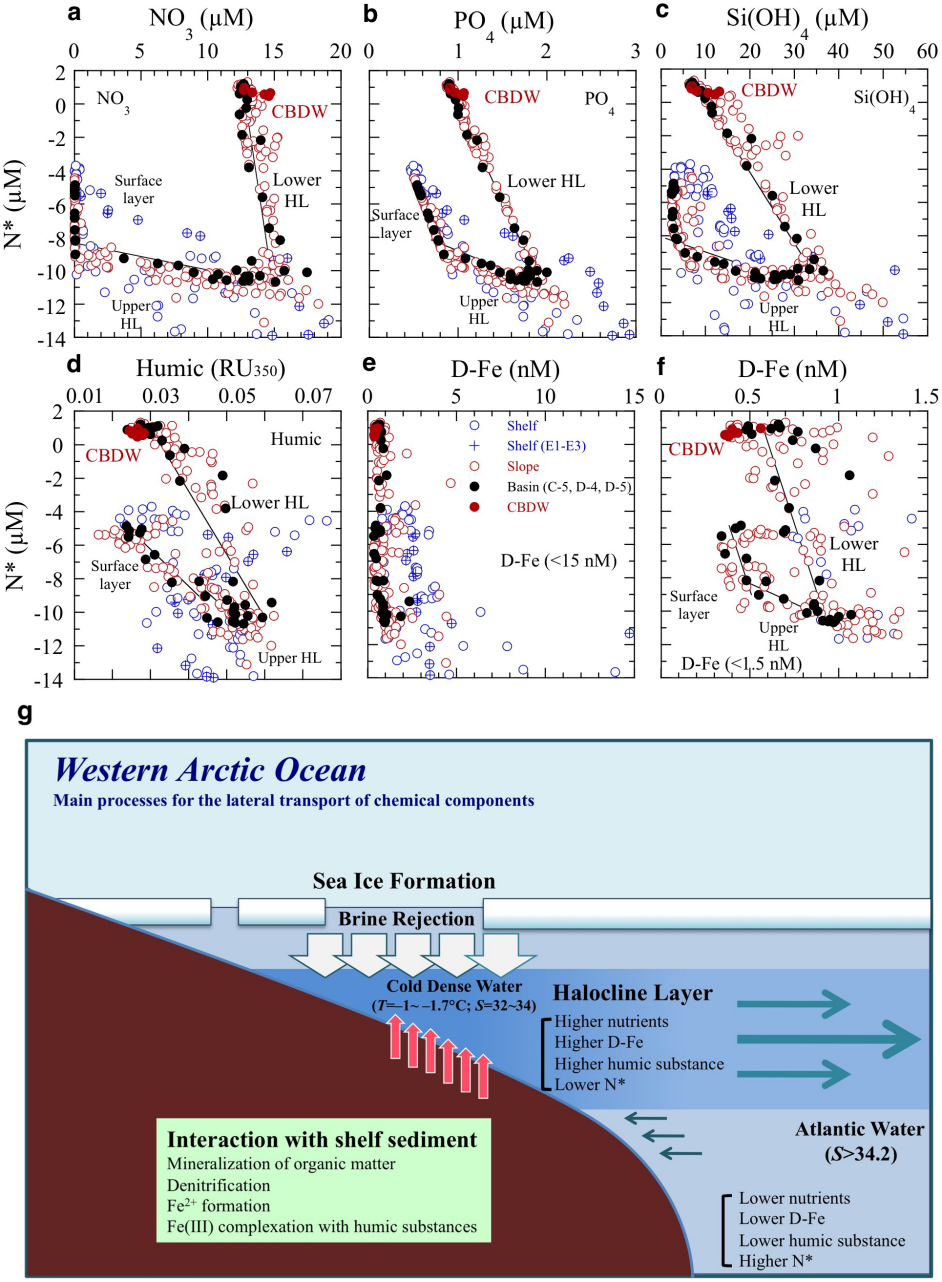
Main processes for the sources and lateral transport of chemical constituents in the halocline layer. Relationships between chemical constituents and N* in the shelf, slope, and basin regions. The solid lines are correlation lines within layers at basin stations (C5, D4, and D5). (a), NO_3_. (b), PO_4_. (c), Si(OH)_4_. (d), humic F-intensity. (e), D-Fe < 15 nM. (f), D-Fe < 1.5 nM. Large negative N* values signal water affected by significant denitrification/anammox within the shelf sediments^22^,^24^. (g), Schematic representation of the three main processes leading to the lateral transport of chemical constituents in the halocline layer of the western Arctic Ocean: (1) brine rejection during sea ice formation; (2) D-Fe, nutrients, and humic DOM supplied from shelf sediments to the overlying brine water in the shelf region; and (3) lateral transport from shelf to basin of D-Fe, nutrients, and humic DOM in the halocline layer.

At the slope and basin stations, the nutrient, humic F-intensity, and D-Fe maximum layer corresponded to N* minimum layer (Figs 2c–g and 4), and their concentrations, especially nutrient, were inversely correlated with N* within layers (surface, upper and lower HL) (Fig. 7). However, the points in the scatterplot of D-Fe against N* are scattered because of the rapid D-Fe supply from the shelf sediments in the shelf regions and the D-Fe removal by particle scavenging during lateral transport from shelf to basin (Fig. 7e,f). The distributions of nutrients, humic F-intensity, and D-Fe in the upper HL were predominantly regulated by the mixing of the N* minimum water with surface water at the interface between the two layers. The distributions in the lower HL were regulated by the mixing of the N* minimum water with the underlying Atlantic water. The minimum (high negative) N* values signal that the water interacted with pore waters within the Chukchi Sea shelf sediments, where notable denitrification/anammox occurs within the shelf sediments^22^,^24^,^26^.

Above the continental shelves of the Bering and Chukchi seas, cold, dense deep and bottom waters are characterized by high levels of nutrients, humic F-intensity, and Fe, and by denitrification/anammox, because of the transport of chemical species across the sediment–water interface during early diagenesis^2^,^6^,^21^,^23^ (Fig. 7g). The elevated D-Fe and T-Fe concentrations in the shelf bottom water result from a marked increase in soluble Fe(II) concentrations in anaerobic pore waters near the sediment–water interface. In fact, we found elevated dissolved Fe(II) concentrations (>0.2 nM) in the cold, dense near-bottom shelf waters of the Chukchi Sea during the T/S *Oshoro-Maru* cruise in summer 2013 (ref. 40). Hypoxic conditions in the water column over the continental shelf lead to an increased flux of reduced Fe(II) from sediments because the oxidation rate of Fe(II) is slowed in the cold, low-oxygen environment^41^,^42^. Although the reduced Fe(II) slowly oxidizes to the less-soluble Fe(III), such as colloidal and particulate Fe(III), in the overlying cold bottom water, Fe(III) complexation with humic DOM would maintain the Fe(III) in the dissolved phase and subsurface D-Fe maxima in the upper HL of the slope and basin regions (Figs 2f,g, 3c,d, and 4).

Our results demonstrate a characteristic, distinct D-Fe maximum, nearly corresponding to the nutrient maximum, in the upper HL along the slope and across the shelf–basin interface in the western Arctic Ocean. Moreover, maximum concentrations of bioactive trace metals (besides Fe), such as Mn, Co, Ni, Cu, Zn, and Cd, have recently been reported in the upper HL of the slope and basin regions in the western Arctic Ocean^43^. The HL, a clear signature of brine resulting from sea ice formation, is generally distributed over the depth range of 50–250 m in the Canada Basin of the western Arctic Ocean. The greater depth extent of the HL in the Canada Basin is also associated with maximum levels of Fe and other chemical constituents^2^,^3^,^7^,^44^. This is consistent with the interpretation that D-Fe-rich and nutrient-rich upper HL water is spreading laterally from the shelf region to the slope and basin regions of the western Arctic Ocean (Fig. 7g). Ecosystems in the Arctic Ocean are being biochemically affected by marine environmental changes accompanying the ongoing rapid reduction in the summer sea ice coverage. In the western Arctic Ocean, the distinctive Fe- and nutrient-rich upper HL contains sufficient D-Fe to allow full utilization of nitrate by phytoplankton (Fig. 5c). Therefore, phytoplankton productivity in the Arctic Ocean can be greatly affected by the upward transport of these subsurface waters enriched in D-Fe and nutrients by eddy and shelf upwelling or by the decrease in the supply in D-Fe and nutrients from the subsurface layer by the increase in sea ice meltwater coverage.

## Methods

### Sample collection and treatment

Samples were collected during cruise MR12E03 of the Japanese R/V *Mira*i between 15 September and 4 October 2012 in the western Arctic Ocean (Fig. 1). The samples were collected in acid-cleaned, Teflon-coated, 10-L Niskin-X sampling bottles with a Teflon sampling spigot (General Oceanics) attached to a CTD-RMS (conductivity-temperature-depth probe-rosette multi-sampler). We collected 12 seawater samples from the water column between 5 and 500 m depth at each station (A1–A3 (C4)–A8 (B1), B2–B4 (D3)–B5, C3, C5, and D4) in the slope and basin regions, 23 samples from the water column between 5 and 3000 m depth at basin station D5, and five to nine samples from the water column between 5 m depth and the sea bottom at each station (C1, C2, D1, D2, and E1–E9) in the shelf region (Fig. 1 and Table 1). Seawater samples were gravity-filtered on deck for analyses of D-Fe and humic F-intensity by connecting a 0.22 µm pore size membrane filter (Durapore cartridge, Millipak 100; Millipore) to the sampling spigot on the Niskin bottles with Teflon and silicon tubing. The filtered (<0.22 µm) and unfiltered seawater samples (100 ml each) used for the D-Fe and T-Fe analyses, respectively, were initially collected in acid-cleaned, 125-ml low-density polyethylene bottles. Then after collection they were acidified with ultrapure HCl to pH 1.7–1.8 in a class 100 clean air bench in a clean room on board the research vessel and allowed to stand at room temperature for at least 3 months until iron analysis in the laboratory. The effect of acidification for this long time storage would have on solubilizing any colloidal Fe phases that might be present in the D-Fe fraction (<0.22 µm pore size), an uncertainty of great concern especially in estuarine and coastal waters, where dissolved Fe concentrations may be higher and colloidal Fe comprises a greater fraction^45^. In addition, the acidification of unfiltered seawater samples for a short time may lead to measure some operational fraction of acid leachable particulate Fe and dissolved Fe fraction. The filtered samples (7–8 ml each) for humic-like fluorescent DOM analysis were collected into 10-ml acrylic tubes (Sanplatec Corp.) and immediately frozen and kept below –20°C in the dark (1–2 months) until measurement in the laboratory. It has been reported that the storage under freezing conditions does not measurably affect the humic-like fluorescence intensity of seawater samples^46^,^47^. All tubing and filters were acid-washed before use.

### Iron analysis

Dissolved Fe and T-Fe were measured by an automated Fe analyzer (Kimoto Electric Co. Ltd.) using a combination of chelating resin concentration and luminol-hydrogen peroxide chemiluminescence (CL) detection in a closed flow-through system as described previously^2^,^18^,^21^,^30^,^48^ and verified against seawater reference materials. Briefly, acidified iron samples were buffered at pH 3.2 with an 8.15 M buffer solution of quartz-distilled formic acid and a 4.54 M ultrapure grade ammonium solution (0.8 ml per 100-ml sample solution) in a class 100 clean-air bench in the laboratory on shore. Iron in each buffered sample was selectively collected on 8-hydroxyquinoline immobilized chelating resin and then eluted with dilute (0.3 M) HCl. The eluent was mixed successively with luminol solution, 0.6 M aqueous ammonia, and 0.7 M H_2_O_2_, and then the mixture was introduced into the CL cell. Finally, the iron concentration was determined from the CL intensity. The accuracy of this analysis was checked using SAFe (Sampling and Analysis of Fe) reference materials (pH 1.7–1.8). The D-Fe concentration in the SAFe surface (S) and deep (D1) intercalibration waters, as determined by our analytical method in the present study after being buffered at pH 3.2, was 0.098 ± 0.005 nM (*n* = 5) for S and 0.70 ± 0.02 nM (*n* = 6) for D1, consistent with the community consensus values of 0.090 ± 0.007 nM for S and 0.67 ± 0.07 nM for D1 (ref. 49).

### Humic-like fluorescent DOM and nutrient analysis

Humic-like fluorescent DOM was quantified by the humic-like fluorescence intensity (humic F-intensity) by a method reported previously^2^,^21^,^30^. The frozen 0.22-µm filtered samples in acrylic tubes were thawed and warmed overnight to room temperature in the dark, and then the humic F-intensity was measured in a 1-cm quartz cell with a Hitachi F-2000 fluorescence spectrophotometer at 320 nm excitation (Ex) and 420 nm emission (Em) wavelengths with a 10-nm bandwidth^46^,^47^. Fluorescence intensity was expressed in terms of quinine sulfate units (1 QSU = 1 ppb quinine sulfate in 0.05 M H_2_SO_4_, excitation 320 nm, emission 420 nm; ref. 50) and then converted to the unified scale of fluorescence Raman units at an excitation wavelength of 350 nm (RU_350_) by using the equation RU_350_ = QSU_320/420_ × 0.012 (refs. 51,52). Nutrient concentrations were measured with a QuAAtro system by Marine Works Japan on behalf of the Japan Marine Science and Technology Center (JAMSTEC) according to “The GO-SHIP Repeat Hydrography Manual (ref. 53)” using the Reference Materials of Nutrients in Seawater. The analytical precisions were estimated to be 0.12% for nitrate, 0.21% for nitrite, 0.19% for phosphate, 0.11% for silicate, and 0.34% for ammonia in terms of median of precision. General descriptions of the R/V *Mirai* cruise MR12E03 were provided in the cruise report, which is already open to the public via the JAMSTEC data website (http://www.godac.jamstec.go.jp/darwin/e).

## Figures and Tables

**Table 1:** Description of stations in the western Arctic Ocean

	Position			
	Latitude	Longitude		
Station	(N)	(W)	Bottom Depth (m)	Sampling date
(Basin region)				
C5	76°00.07′	174°01.00′	2,137	18.Sep.12
D4	73°49.22′	156°41.15′	3,684	29.Sep.12
D5	74°49.87′	154°00.40′	3,851	29.Sep.12
(Slope region)				
A1	75°23.40′	177°50.67′	721	19.Sep.12
A2	75°26.70′	175°50.38′	610	19.Sep.12
A3 (C4)	75°21.45′	172°58.22′	453	17.Sep.12
A4	74°99.97′	170°00.78′	262	20.Sep.12
A5	74°99.93′	168°00.15′	167	20.Sep.12
A6	74°99.95′	166°00.43′	487	20.Sep.12
A7	74°99.97′	164°00.12′	690	22.Sep.12
A8 (B1)	75°00.43′	161°89.79′	1,987	20.Sep.12
B2	74°16.92′	162°33.32′	984	23.Sep.12
B3	73°33.47′	160°02.08′	1,390	23.Sep.12
B4 (D3)	72°86.53′	157°96.47′	1,573	30.Sep.12
B5	72°50.22′	156°00.63′	1,896	24.Sep.12
C3	74°67.02′	170°92.47′	233	17.Sep.12
(Shelf region)				
C1	72°24.00′	168°73.87′	55	16.Sep.12
C2	73°50.17′	168°74.68′	118	16.Sep.12
D1	72°00.02′	159°99,72′	30	30.Sep.12
D2	72°49.98′	158°79.88′	55	30.Sep.12
E1	67°75.00′	168°75.00′	51	4.Oct.12
E2	67°99.87′	168°74.90′	59	3.Oct.12
E3	68°25.03′	168°74.92′	57	3.Oct.12
E4	69°00.05′	168°75.25′	53	15.Sep.12
E5	70°74.98′	168°74.68′	37	15.Sep.12
E6	70°75.12′	167°99.63′	48	2.Oct.12
E7	70°74.92′	165°99.95′	40	1.Oct.12
E8	70°74.98′	163°99.58′	47	1.Oct.12
E9	70°74.98′	161°99.43′	43	1.Oct.12
